# Differences in characteristics between healthcare-associated and community-acquired infection in community-onset *Klebsiella pneumoniae* bloodstream infection in Korea

**DOI:** 10.1186/1471-2334-12-239

**Published:** 2012-10-03

**Authors:** Younghee Jung, Myung Jin Lee, Hye-Yun Sin, Nak-Hyun Kim, Jeong-Hwan Hwang, Jinyong Park, Pyoeng Gyun Choe, Wan Beom Park, Eu Suk Kim, Sang-Won Park, Kyoung Un Park, Hong Bin Kim, Nam-Joong Kim, Eui-Chong Kim, Kyoung-Ho Song, Myoung-don Oh

**Affiliations:** 1 Department of Internal Medicine, Seoul National University Bundang Hospital, 173 Gumi-ro, Bundang-gu, Seongnam, 463-707, Republic of Korea; 2 Department of Laboratory Medicine, Seoul National University Bundang Hospital, Seongnam, Korea; 3 Department of Internal Medicine, Seoul National University Hospital, Seoul, Korea; 4 Department of Laboratory Medicine, Seoul National University Hospital, Seoul, Korea; 5 Seoul National University College of Medicine, Seoul, Korea

**Keywords:** Klebsiella pneumoniae, Bacteremia, Community-acquired infections, Healthcare-associated, Community-onset infection, Epidemiology

## Abstract

**Background:**

Healthcare-associated (HCA) infection has emerged as a new epidemiological category. The aim of this study was to evaluate the impact of HCA infection on mortality in community-onset *Klebsiella pneumoniae* bloodstream infection (*Kp*BSI).

**Methods:**

We conducted a retrospective study in two tertiary-care hospitals over a 6-year period. All adult patients with *Kp*BSI within 48 hours of admission were enrolled. We compared the clinical characteristics of HCA and community-acquired (CA) infection, and analyzed risk factors for mortality in patients with community-onset *Kp*BSI.

**Results:**

Of 553 patients with community-onset *Kp*BSI, 313 (57%) were classified as HCA- *Kp*BSI and 240 (43%) as CA-*Kp*BSI. In patients with HCA-*Kp*BSI, the severity of the underlying diseases was higher than in patients with CA-*Kp*BSI. Overall the most common site of infection was the pancreatobiliary tract. Liver abscess was more common in CA-*Kp*BSI, whereas peritonitis and primary bacteremia were more common in HCA-*Kp*BSI. Isolates not susceptible to extended-spectrum cephalosporin were more common in HCA- *Kp*BSI than in CA-*Kp*BSI (9% [29/313] vs. 3% [8/240]; *p =* 0.006). Overall 30-day mortality rate was significantly higher in HCA-*Kp*BSI than in CA-*Kp*BSI (22% [70/313] vs*.* 11% [27/240]; *p* = 0.001). In multivariate analysis, high Charlson’s weighted index of co-morbidity, high Pitt bacteremia score, neutropenia, polymicrobial infection and inappropriate empirical antimicrobial therapy were significant risk factors for 30-day mortality.

**Conclusions:**

HCA-*Kp*BSI in community-onset *Kp*BSI has distinctive characteristics and has a poorer prognosis than CA-*Kp*BSI, but HCA infection was not an independent risk factor for 30-day mortality.

## Background

Within the last decade, the concept of healthcare-associated (HCA) infection has been introduced, and HCA infection has been described as an epidemiological category different from both community-acquired (CA) and nosocomial infection [1,2]. Most importantly, mortality in HCA infection seems to be generally higher than that in CA infection, and similar to that in nosocomial infection [2-4]. However, there are conflicting results regarding whether HCA infection is an independent risk factor for mortality in bloodstream infection [1,5]. A few pathogens have been studied in terms of HCA infection with *S. aureus* dominating the research, and these studies reported inconsistent data concerning the impact of HCA infection on mortality [6-13]. For gram-negative bacteria, the data on the impact of HCA infection on mortality were conflicting, as well [9-12].

*Klebsiella pneumoniae* is one of the most important gram-negative bacteria clinically, and *K. pneumoniae* bloodstream infection (*Kp*BSI) has a mortality rate of about 20% [11,14-16]. Classically, *Kp*BSI was simply classified into CA and nosocomial infections depending on bacteremia onset time: within 48 hours and after 48 hours of admission, respectively, and the different characteristics of CA-*Kp*BSI versus nosocomial *Kp*BSI have been well evaluated [11,14,15,17,18]. CA-*Kp*BSI is usually associated with liver abscesses in patients with diabetes in East Asian countries, such as Korea and Taiwan [19-22]. On the other hand, nosocomial *Kp*BSI presents as primary bacteremia and/or pneumonia in patients with severe underlying diseases like malignancies. Thus, nosocomial infection has a higher mortality than CA infection [11,14,15,17,18]. However, there have been few studies of HCA-*Kp*BSI [11-13]. Therefore, we aimed to evaluate the impact of HCA infection on mortality and to compare the clinical characteristics of HCA and CA infection in patients with community-onset *Kp*BSI.

## Methods

### Study setting and patients

We conducted a retrospective study of the medical records of the patients with *Kp*BSI from January 2003 to December 2008 at Seoul National University Hospital (a 1,600-bed tertiary-care hospital, Seoul, Korea) and Seoul National University Bundang Hospital (a 900-bed tertiary-care hospital, Seongnam, Korea). All adult patients (≥18 year) who had *Kp*BSI within 48 hours of admission were enrolled. When there were multiple *Kp*BSI episodes only the first was included. We collected patient’s data on age, sex, underlying disease, site of infection, laboratory findings, microbiologic characteristics and treatment outcomes. To assess treatment outcomes we investigated 30-day mortality. This study was approved by the institutional review board of Seoul National University Hospital (IRB No. H-1010-062-336) and Seoul National University Bundang Hospital (IRB No. B-0910/086-004) according to the Helsinki Declaration.

### Definitions

Community-onset *Kp*BSI was defined as *Kp*BSI occurring within 48 hours of admission. HCA-*Kp*BSI was defined when a patient had one of the following medical histories: (1) intravenous therapy at home or in an outpatient clinic within the previous 30 days; (2) renal dialysis in a hospital or clinic within the previous 30 days; (3) hospitalization for 2 or more days within the previous 90 days; (4) residence in a nursing home or long-term care facility for 2 or more days [1]. Patients without any of these factors were classified as CA-*Kp*BSI.

Biliary tract disease was defined when one or more complicated biliary stone was present, or there was a structural biliary abnormality due to benign or malignant disease. Chronic liver disease referred to chronic hepatitis and liver cirrhosis due to any cause. Charlson’s weighted index of co-morbidity and the Pitt bacteremia score were used to evaluate the severities of underlying disease and of acute illness, respectively [23,24]. Shock was defined as a decrease in systolic blood pressure to 90mmHg or less, or a decrease of at least 40mmHg below baseline blood pressure despite adequate fluid resuscitation [25]. An absolute neutrophil count of less than 500/mL was defined as neutropenia. Polymicrobial infection was defined when any pathogen other than *K. pneumoniae* was isolated from blood culture at the same time as the *K. pneumoniae*, and the isolated pathogen also had clinical significance. Infection focus was assessed clinically by attending physician in accordance with site of isolation of *K. pneumoniae.*

The empirical antimicrobial therapy was defined as the initial antibiotic choice before the results of blood culture and antimicrobial susceptibility tests were available, and the definitive antimicrobial therapy was defined as the antibiotic choice after the report of microbiologic tests. *K. pneumoniae* which was not susceptible to either cefotaxime or ceftazidime was considered as ‘suspected extended-spectrum beta-lactamase (ESBL) producing *K. pneumoniae*’. Antimicrobial therapy was considered as ‘inappropriate’ when the treatment regimen did not include any antibiotic active *in vitro*. In addition 3^rd^ generation cephalosporin monotherapy for ‘suspected ESBL-producing *K. pneumoniae*’ was considered ‘inappropriate’, regardless of the results of antibiotic susceptibility tests.

### Microbiological analysis

All isolates were defined by BacT/ALERT FA and FN (bioMe´rieux, Durham, North Carolina). Antimicrobial susceptibility was identified by disk diffusion tests from 2003 to 2006, and by Microscan WalkAway-96 (Siemens Healthcare Diagnostics, Deerfield, Illinois) from 2007 to 2008, using the criteria of the Clinical and Laboratory Standards Institute (CLSI; formerly, National Committee for Clinical Laboratory Standards) guidelines. For the available ‘suspected ESBL-producing *K. pneumoniae*’ isolates, ESBL production was determined by the double disk synergy test according to the CLSI performance standards [26].

### Statistical analysis

Student’s *t*-test was used to compare continuous variables and the χ^2^ test or Fisher’s exact test was used to compare categorical variables. To identify independent risk factors for 30-day mortality, a stepwise logistic regression model was used. Risk factors with a *p* value <0.10 in the univariate analysis for 30-day mortality were included in the initial model, and forward stepwise selection was performed to develop the final model. We included the Pitt bacteremia score instead of shock and, the Charlson’s weighted index of co-morbidity instead of underlying diseases to avoid data overlap in the multivariate analysis. *p* <0.05 was considered statistically significant. PASW for Windows (version 18 software package; SPSS Inc., Chicago, IL, USA) was used for all analyses.

## Results

### Demographics and underlying diseases

592 patients with community-onset *Kp*BSI were identified in the 6-year period. Of these, 553 (93%) were analyzed, because 34 patients were lost to follow-up and medical record was not available in 5 patients. Of the 553 patients with community-onset *Kp*BSI, 313 (57%) were classified as HCA-*Kp*BSI and 240 (43%) as CA-*Kp*BSI. The mean age of the 553 patients was 61 years (median: 63 years, range: 18–103), and 348 (63%) of the patients were male. Solid tumor was the most common underlying disease (238 patients, 43%). 145 (26%) patients had diabetes mellitus and 123 (22%) had chronic liver disease.

The demographics and underlying diseases of HCA-*Kp*BSI and CA-*Kp*BSI are listed in Table 1. Solid tumor (58% vs*.* 24%; *p* <0.001), hematologic malignancy (7% vs*.* 2%; *p* = 0.002) and chronic liver disease (27% vs*.* 16%; *p =* 0.003) were more common in HCA-*Kp*BSI than in CA-*Kp*BSI. Diabetes mellitus (31% vs*.* 23%; *p =* 0.031) was more common in CA-*Kp*BSI than in HCA-*Kp*BSI.

**Table 1:** **Clinical characteristics of community-acquired and healthcare-associated infections in patients with community-onset*Klebsiella pneumoniae*bloodstream infection**

	**CA-*Kp*BSI**	**HCA-*Kp*BSI**	***p***
	**(n = 240)**	**(n = 313)**	
Age (years) (mean ±SD)	63.0 (±13.6)	60.3 (±12.9)	0.015
Male sex	142 (59.2)	206 (65.8)	0.124
Underlying disease			
Diabetes mellitus	74 (30.8)	71 (22.7)	0.031
Chronic liver disease	39 (16.3)	84 (26.8)	0.003
Biliary tract disease	42 (17.5)	43 (13.7)	0.224
Chronic kidney disease	13 (5.4)	19 (6.1)	0.774
Respiratory disease	18 (7.5)	21 (6.7)	0.719
Solid tumor	58 (24.2)	180 (57.5)	<0.001
Hematologic malignancy	4 (1.7)	23 (7.3)	0.002
Solid organ transplantation	5 (2.1)	9 (2.9)	0.557
Charlson’s WIC (≥3)	47 (19.6)	141 (45.0)	<0.001
Primary infection site			
Urinary tract	31 (12.9)	34 (10.9)	0.457
Peritoneum	11 (4.6)	46 (14.7)	<0.001
Pancreatobiliary tract	82 (34.2)	102 (32.6)	0.696
Liver	67 (27.9)	41 (13.1)	<0.001
Lung	16 (6.7)	28 (8.9)	0.326
Skin and soft tissue	4 (1.7)	8 (2.6)	0.477
Bone	4 (1.7)	2 (0.6)	0.411
Central nervous system	1 (0.4)	0 (0)	0.434
Others^a^	1 (0.4)	3 (1.0)	0.637
Unknown	21 (8.8)	52 (16.6)	0.007
Metastatic infection	11 (4.6)	9 (2.9)	0.286
Endophthalmitis	5 (2.1)	3 (1.0)	0.303
Pitt bacteremia score (≥4)	36 (15.0)	56 (17.9)	0.366
Shock at presentation	55 (22.9)	101 (32.3)	0.015
Neutropenia at presentation	3 (1.3)	42 (13.4)	<0.001
Polymicrobial infection	34 (14.2)	47 (15.0)	0.780
Initial antimicrobial regimen			
Piperacillin-tazobactam	9 (3.8)	30 (9.6)	0.008
Quinolone	23 (9.6)	18 (5.8)	0.088
1^st^ generation cephalosporin	2 (0.8)	0 (0)	0.188
3^rd^ generation cephalosporin	173 (72.1)	186 (59.4)	0.002
Carbapenem	30 (12.5)	48 (15.3)	0.342
Inappropriate empirical antimicrobial therapy	14 (5.8)	26 (8.3)	0.266
Inappropriate definitive antimicrobial therapy^b^	7/223 (3.1)	7/278 (2.5)	0.675
30-day mortality	27 (11.3)	70 (22.4)	0.001

Data indicate no. (%) of patients. WIC, weighted index of co-morbidity; CA-*Kp*BSI, community-acquired *Klebsiella pneumoniae* bloodstream infection; HCA-*Kp*BSI, healthcare-associated *Klebsiella pneumoniae* bloodstream infection. ^a^ Others were one appendicitis, one pericarditis and two periodontitis. ^b^ 52 patients were excluded from the analysis (15 patients were transferred to other hospitals and 37 died before the culture results were available).

### Primary sites of infection and treatment outcomes

The pancreatobiliary tract was the most common site of infection (184 cases, 33%). Liver (108, 20%) and primary bacteremia (unknown focus) (73, 13%) were also frequent sites of infection. Initially 156 (28%) patients presented shock and 45 (8%) had neutropenia. 40 (7%) patients of the 553 patients were treated with inappropriate empirical antimicrobial therapy. After excluding 52 of the 553 patients (15 were transferred to other hospitals and 37 died before the culture results were reported), 14 of the remaining 501 patients (3%) were found to have been treated with inappropriate definitive antimicrobial therapy.

The primary sites of infection and treatment outcomes of HCA-*Kp*BSI and CA-*Kp*BSI are also compared in Table 1. Peritonitis (15% vs*.* 5%; *p* <0.001) and primary bacteremia (17% vs*.* 9%; *p =* 0.007) were more common in HCA-*Kp*BSI than in CA-*Kp*BSI. On the other hand, liver abscess (28% vs*.* 13%; *p* <0.001) was more frequent in CA-*Kp*BSI than in HCA-*Kp*BSI. Initial shock (32% vs*.* 23%; *p =* 0.015) and neutropenia (13% vs*.* 1%; *p* <0.001) were more common in HCA-*Kp*BSI than in CA-*Kp*BSI. The 30-day mortality of HCA-*Kp*BSI was higher than that of CA-*Kp*BSI (22% vs*.* 11%; *p =* 0.001).

### Antimicrobial susceptibility

48 of the 553 isolates (9%) were not susceptible to ciprofloxacin and 37 isolates (7%) were not susceptible to either cefotaxime or ceftazidime. Of these 37 isolates, 27 were available for ESBL confirmatory tests and we performed the double disk synergy test on them. Eighteen were confirmed as producing ESBL. The antimicrobial susceptibilities of HCA-*Kp*BSI and CA-*Kp*BSI are compared in Table 2. A significantly higher proportion of the HCA-*Kp*BSI than of the CA-*Kp*BSI was resistant to tested antimicrobial agents other than imipenem and amikacin.

**Table 2:** **Comparison of the antimicrobial susceptibility of community-acquired (CA) and healthcare-associated (HCA)*Klebsiella pneumoniae*bloodstream infection (*Kp*BSI)**

**Antibiotics**	**CA-*Kp*BSI (n = 240)**	**HCA-*Kp*BSI (n = 313)**	***p***
	**(Non-susceptible/T (%))**	**(Non-susceptible/T (%))**	
Ciprofloxacin	12/240 (5.0)	36/313 (11.5)	0.007
Extended-spectrum cephalosporin	8/240 (3.3)	29/313 (9.3)	0.006
Cefotaxime	8/240 (3.3)	25/313 (8.0)	0.022
Ceftazidime	6/240 (2.5)	25/313 (8.0)	0.005
ESBL-production^a^	2/237 (0.8)	16/310 (5.2)	0.006
Piperacillin plus tazobactam	6/239 (2.5)	19/311 (6.1)	0.045
Aztreonam	5/175 (2.9)	26/253 (11.1)	0.004
Imipenem	1/240 (0.4)	1/313 (0.3)	1.000
Amikacin	7/240 (2.9)	17/313 (5.4)	0.150
Gentamicin	6/240 (2.5)	22/313 (7.0)	0.016
Tobramycin	7/177 (4.0)	26/253 (10.3)	0.015

Data indicate number of non-susceptible isolates/total number of tested isolates (%).

T, total number of tested isolates; ESBL, extended-spectrum beta-lactamase.

^a^ 5 isolates among the CA-*Kp*BSI were available for ESBL confirmatory tests and 14 among the HCA-*Kp*BSI.

### Risk factors for 30-day mortality

The results of the univariate analyses of risk factors for 30-day mortality are shown in Table 3. High Charlson’s weighted index of co-morbidity was a risk factor (odds ratio [OR], 2.86; 95% confidence interval [CI], 1.83-4.48) and, when we analyzed each underlying disease, solid tumor (OR, 3.32; 95% CI, 2.09-5.28) and hematologic malignancy (OR, 3.52; 95% CI, 1.58-7.84) also turned out to be significant risk factors. Infections of unknown origin (OR, 3.71; 95% CI, 2.17-6.34) and respiratory infections (OR, 3.38; 95% CI, 1.76-6.48) developed more frequently in non-survivors than in survivors. In contrast, liver abscess (OR, 0.11; 95% CI, 0.03-0.35) and pancreatobiliary infection (OR, 0.50; 95% CI, 0.30-0.84) were more common in survivors than in non-survivors. In addition, high Pitt bacteremia score (OR, 8.04; 95% CI, 4.87-13.28), neutropenia at initial presentation (OR, 4.48; 95% CI, 2.37-8.46), inappropriate empirical antimicrobial therapy (OR, 2.46; 95% CI, 1.22-4.96), polymicrobial infection (OR, 2.30; 95% CI, 1.34-3.94) and healthcare-associated infection (OR, 2.27; 95% CI, 1.41-3.68) were risk factors in univariate analyses. There was no significant difference in rates of antimicrobial resistance to ciprofloxacin (7.0% in survivors vs. 9.3% in non-survivors; *p* = 0.440) and extended-spectrum cephalosporin (6.4% in survivors vs. 8.2% in non-survivors; *p* = 0.499) between survivors and non-survivors.

**Table 3:** **Risk factors for 30-day mortality among patients with community-onset*Klebsiella pneumoniae*bloodstream infection in univariate analysis**

	**No. of survivors**	**No. of non-survivors**	**OR (95% CI)**	***p***
	**(n = 456)**	**(n = 97)**		
Age (year) (mean ±SD)	61.1 (±13.5)	63.3 (±12.1)		0.146
Male sex	287 (62.9)	61 (62.9)	1.00 (0.63-1.57)	0.992
Underlying disease				
Diabetes mellitus	128 (28.1)	17 (17.5)	0.55 (0.31-0.96)	0.032
Chronic liver disease	94 (20.6)	29 (29.9)	1.64 (1.01-2.68)	0.046
Biliary tract disease	77 (16.9)	8 (8.2)	0.44 (0.21-0.95)	0.032
Chronic kidney disease	28 (6.1)	4 (4.1)	0.66 (0.26-1.92)	0.440
Respiratory disease	34 (7.5)	5 (5.2)	0.68 (0.26-1.77)	0.421
Solid tumor	173 (37.9)	65 (67.0)	3.32 (2.09-5.28)	<0.001
Hematologic malignancy	16 (3.5)	11 (11.3)	3.52 (1.58-7.84)	0.003
Solid organ transplantation	13 (2.9)	1 (1.0)	0.36 (0.05-2.75)	0.482
Charlson's WIC (≥3)	135 (29.6)	53 (54.6)	2.86 (1.83-4.48)	<0.001
Primary infection site				
Urinary tract	58 (12.7)	7 (7.2)	0.53 (0.24-1.22)	0.126
Peritoneum	42 (9.2)	15 (15.5)	1.80 (0.96-3.40)	0.066
Pancreatobiliary tract	163 (35.7)	21 (21.6)	0.50 (0.30-0.84)	0.007
Liver	105 (23.0)	3 (3.1)	0.11 (0.03-0.35)	<0.001
Lung	27 (5.9)	17 (17.5)	3.38 (1.76-6.48)	<0.001
Skin and soft tissue	8 (1.8)	4 (4.1)	2.41 (0.71-8.17)	0.239
Bone	5 (1.1)	1 (1.0)	0.94 (0.11-8.13)	1.000
Central nervous system	1 (0.2)	0 (0)	0.82 (0.79-0.86)	1.000
Others^a^	3 (0.7)	1 (1.0)	1.57 (0.16-15.28)	0.539
Unknown	45 (9.9)	28 (28.9)	3.71 (2.17-6.34)	<0.001
Metastatic infection	18 (3.9)	2 (2.1)	0.51 (0.12-2.25)	0.551
Endophthalmitis	8 (1.8)	0 (0)	0.82 (0.79-0.86)	0.362
Pitt bacteremia score (≥4)	46 (10.1)	46 (47.4)	8.04 (4.87-13.28)	<0.001
Shock at presentation	97 (21.3)	59 (60.8)	5.75 (3.61-9.15)	<0.001
Neutropenia at presentation	25 (5.5)	20 (20.6)	4.48 (2.37-8.46)	<0.001
Healthcare-associated infection	243 (53.3)	70 (72.2)	2.27 (1.41-3.68)	0.001
Polymicrobial infection	57 (12.5)	24 (24.7)	2.30 (1.34-3.94)	0.002
Antimicrobial resistance				
non-susceptible to CIP	32 (7.0)	9 (9.3)	1.36 (0.63-2.94)	0.440
non-susceptible to ESC	29 (6.4)	8 (8.2)	1.32 (0.59-2.99)	0.499
Inappropriate empirical antimicrobial therapy	27 (5.9)	13 (13.4)	2.46 (1.22-4.96)	0.010
Inappropriate definitive antimicrobial therapy^b^	12/446 (2.7)	2/55 (3.6)	1.37 (0.30-6.26)	0.659

Data indicate no. (%) of patients.

WIC, weighted index of co-morbidity; CIP, ciprofloxacin; ESC, extended-spectrum cephalosporin; OR, odds ratio; CI, confidence interval.

^a^ Others were one appendicitis, one pericarditis and two periodontitis.

^b^ 52 patients were excluded from the analysis (15 patients were transferred to other hospitals and 37 died before the culture results were available).

From the multivariate logistic regression analysis, significant risk factors for 30-day mortality were high (≥3) Charlson’s weighted index of co-morbidity (adjust odds ratio [aOR], 3.23; 95% CI, 1.88-5.57), high (≥4) Pitt bacteremia score (aOR, 8.43; 95% CI, 4.70-15.11), neutropenia (aOR, 2.60; 95% CI, 1.24-5.48), polymicrobial infection (aOR, 2.36; 95% CI, 1.21-4.60) and inappropriate empirical antimicrobial therapy (aOR, 2.43; 95% CI, 1.07-5.52). Liver abscess (aOR, 0.17; 95% CI, 0.05-0.58) and pancreatobiliary tract infection (aOR, 0.42; 95% CI, 0.23-0.79) were found to be protective factors (Table 4). HCA infection was not an independent risk factor for mortality in multivariate analysis (aOR, 1.27; 95% CI, 0.70-2.30).

**Table 4:** **Significant risk factors for 30-day mortality among community-onset*Klebsiella pneumoniae*bloodstream infection in multivariate analysis**

	**No. of survivors (n = 456)**	**No. of non-survivors (n = 97)**	**Adjusted OR (95% CI)**	***p***
Charlson's WIC (≥3)	135 (29.6)	53 (54.6)	3.23 (1.88-5.57)	<0.001
Pitt bacteremia score (≥4)	46 (10.1)	46 (47.4)	8.43 (4.70-15.11)	<0.001
Neutropenia	25 (5.5)	20 (20.6)	2.60 (1.24-5.48)	0.012
Polymicrobial infection	57 (12.5)	24 (24.7)	2.36 (1.21-4.60)	0.012
Pancreatobiliary infection	163 (35.7)	21 (21.6)	0.42 (0.23-0.79)	0.006
Liver abscess	105 (23.0)	3 (3.1)	0.17 (0.05-0.58)	0.038
Inappropriate empirical antimicrobial therapy	27 (5.9)	13 (13.4)	2.43 (1.07-5.52)	0.035

Data indicate no. (%) of patients.

WIC, weighted index of co-morbidity; OR, odds ratio; CI, confidence interval.

## Discussion

In a previous study, we demonstrated that nosocomial *Kp*BSI was different from CA- *Kp*BSI [18]. However, the healthcare system has changed dramatically and this simple dichotomy is no longer appropriate for *Kp*BSI in the current clinical setting. In this study we showed that HCA-*Kp*BSI accounted for over 50% of community-onset *Kp*BSI and HCA-*Kp*BSI had different clinical characteristics from CA-*Kp*BSI in terms of underlying disease, infection focus, antimicrobial susceptibility and treatment outcome. To our knowledge, this is the largest multicenter study comparing the clinical characteristics of HCA-*Kp*BSI and CA-*Kp*BSI [11,12].

Cancer was the most common associated condition in HCA-*Kp*BSI. In contrast, diabetes mellitus was the most common associated condition in CA-*Kp*BSI. The distribution of underlying disease in HCA-*Kp*BSI was similar to that in nosocomial *Kp*BSI, except for the frequency of chronic liver disease. While we found previously that the frequency of this disease did not differ between CA-*Kp*BSI and nosocomial *Kp*BSI [18], it was more common in HCA-*Kp*BSI than in CA-*Kp*BSI (27% vs*.* 16%; *p* = 0.003) in the present study. The latter finding is similar to that of a study performed in Taiwan, although in the Taiwanese study the difference was not statistically significant (liver cirrhosis in HCA-*Kp*BSI [14.0%] vs. CA- *Kp*BSI [10.6%]; *p* = 0.339) [12].

The primary site of infection was identified in 87% of community-onset *Kp*BSI. The most common source was the pancreatobiliary tract (33%), followed by liver abscess (20%). Liver abscess was more frequent in CA-*Kp*BSI than in HCA-*Kp*BSI. Compared to nosocomial *Kp*BSI, in which liver abscess was very rare (0% to 2%) [11,14,18], HCA-*Kp*BSI was quite frequently associated with liver abscess (13%). Peritonitis was fairly frequent (>10%), more so in HCA-*Kp*BSI than in CA-*Kp*BSI in our analysis; in contrast Wu *et al*. found only a few (<5%) of intra-abdominal infection and no difference in frequency between HCA-*Kp*BSI and CA-*Kp*BSI [12]. Our higher frequency of peritonitis may be due to the prevalence of chronic liver disease caused by hepatitis B or C virus in Korea, which increases the occurrence of spontaneous bacterial peritonitis [27,28].

More of the HCA-*Kp*BSI isolates than of the CA-*Kp*BSI isolates were resistant to antimicrobial agents. Over 10% of the former were not susceptible to ciprofloxacin and 9% were not susceptible to one of the extended-spectrum cephalosporin. Although frequent antimicrobial resistance could affect the inadequacy of the initial choice of antimicrobial agent, there was no difference in rate of inappropriate empirical antimicrobial therapy between the HCA-*Kp*BSI and the CA-*Kp*BSI (6% vs. 8%; *p* = 0.266). This result could have arisen because in cases of healthcare-associated infection clinicians may have taken into account frequencies of antimicrobial resistance when selecting the initial antibiotic. Actually, fewer HCA-*Kp*BSI than CA-*Kp*BSI (6% vs. 10%; *p* = 0.088) were started on quinolones while more were started on piperacillin-tazobactam (Table 1).

Regarding empirical treatment, the proportion of patients treated inappropriately (7.2% of total patients) was much lower than was observed in other studies, which showed that over 20% of patients were treated inappropriately [9,29,30]. This discrepancy might have been the result of differences in the definition of ‘appropriate empirical treatment’, because the definition we used was less strict than those in other studies [31-33]. In addition, broad-spectrum antimicrobial agents, such as 3^rd^ generation cephalosporins or carbapenems, were frequently used empirically in our study (84.6% in CA infection, 74.7% in HCA infection). Considering that only 3.3% of organisms in CA infection and 9.3% of organisms in HCA infection were non-susceptible to extended-spectrum cephalosporins, the use of broad-spectrum antimicrobial agents also might have influenced the lower proportion of patients with treated inappropriately. However, other East Asian studies of *K. pneumoniae* bacteremia also demonstrated a similar proportion of patients treated with inappropriate empirical therapy [12,14,18].

There was a significant difference of 30-day mortality rate between HCA-*Kp*BSI and CA-*Kp*BSI in this study (22% vs. 11%; *p* = 0.001). High Charlson’s weighted index of co-morbidity (≥3), high Pitt bacteremia score (≥4), neutropenia, polymicrobial infection and inappropriate empirical antimicrobial therapy were found to be independent risk factors for mortality. However, HCA infection itself was not a significant risk factor for 30-day mortality in multivariate analysis. This finding is consistent with the recent report from Taiwan and a bloodstream infection study dealing with gram-negative bacteria [9,12]. There are several explanations for this result. First, in our study, underlying disease and acute illness, which are classical risk factors for outcome of infectious disease, may have been so severe as to have attenuated the effect of HCA infection on mortality [23,24,34]. Second, whether the infection focus was removable, and was or was not removed, may have affected mortality more than whether the infection was HCA or not [35]. Liver abscess and pancreatobiliary infection can be classified as infections with removable foci, as opposed to pneumonia or primary bacteremia. In our study, percutaneous or internal drainage was performed in cases of liver abscess and obstructive pancreatobiliary infection, and these kinds of infection were found to be independent protective factors for mortality, as in the previous studies [11,14,18]. Third, as indicated by Friedman *et al*., the definition of HCA infection which we used in this study may have been excessively broad since the definition was based on the U.S. medical system [1]. Unlike the U.S., South Korea has started a national health insurance system in 1977 and extended it nationwide in 1982. Consequently, there is a tendency for more people to access the medical system and be classified as HCA infection in Korea. Such national differences in healthcare systems could complicate the unambiguous identification of patients with HCA infections so as to be able to evaluate the actual effect of HCA infection on mortality. Therefore we need further studies using a more accurate and consistent definition of HCA infection that accords better with variations in clinical practice.

Our study had several limitations. First, there was a potential bias because it was performed retrospectively. Second, it was conducted in tertiary-care and university-affiliated hospitals and there was a large proportion of cancer patients in both the CA-*Kp*BSI and HCA-*Kp*BSI groups. Hence, we cannot extrapolate our result to community-based institutions. Third, we did not evaluate the ‘attributable’ mortality due to *Kp*BSI, so that some of the deaths in our study may not have been related to *Kp*BSI. However, efforts to designate outcomes as ‘attributable’ to infection are often subjective and inconsistent. We therefore employed an unambiguous definition, namely 30-day mortality rate, for evaluating treatment outcomes. Fourth, because we did not review the patients’ previous exposure to antimicrobial agents, we could not determine the influence of that factor on the acquiring resistant organisms and treatment outcome. Additionally, we did not collect data on the variation of antimicrobial therapy, such as duration or dosage; therefore, we could not take into account these issues, which could affect the analysis of risk factors for mortality. However, upon examining the mortality rate in other studies, our data are comparable; therefore, the regimen and duration of therapy we used were also likely to be similar to others [12,14].

What clinicians actually want to know is that HCA infection needs a specific work-up process or treatment. Accordingly, our study can provide useful information. First, we could see the difference of infection focus according to the epidemiological category more clearly by separating HCA infection and CA infection in comparison to our previous report [18]. Peritonitis is more commonly associated with HCA infection than with CA infection in the present study, while the frequency of peritonitis in CA infection was relatively high (20.4%) and was not different from that observed in nosocomial infection in the previous study [18]. However, when we classified more precisely we could see that peritonitis occurred in much lower frequency in true CA infection. In addition, we could identify the infection focus of all but 8.8% of the patients with true CA infection (in previously defined CA infection, 25.7% patients were unidentified for infection focus) [18]. Second, comparing the resistance rate to antimicrobial agents in HCA infection with CA infection can help clinicians choose an initial antimicrobial agent in treating patients of each subset, which means not only should we consider broad-spectrum antimicrobial agents in HCA infection but also that we may not need to start broad-spectrum antimicrobial agents, such as extended-spectrum cephalosporins or carbapenems, in CA infection. Based on our data, quinolone can be a drug of choice in treating true CA-*Kp*BSI. Finally, even though we showed many differences between HCA infection and CA infection, we did not find HCA infection to be an independent risk factor for mortality in *Kp*BSI, which confirmed that the already known risk factors for mortality (severity of underlying disease, inadequate empirical therapy and severity of acute illness) are more important predictors of mortality in *Kp*BSI [12,14].

## Conclusion

HCA-*Kp*BSI represented over half of community-onset *Kp*BSI and had different characteristics from CA-*Kp*BSI. In HCA-*Kp*BSI, underlying diseases were more severe, primary bacteremia and peritonitis were more common and resistance to antimicrobials was more frequent than in CA-*Kp*BSI. HCA-*Kp*BSI had higher 30-day mortality than CA-*Kp*BSI, but HCA infection was not an independent risk factor for 30-day mortality. In present-day clinical circumstances, HCA-*Kp*BSI should be identified as a distinctive category and be approached in a different way from CA-*Kp*BSI.

## Competing interests

There are no potential conflicts of interest for any authors.

## Authors’ contribution

All authors conceived of the study. YHJ, MJL, HYS collected the data. KHS and YHJ carried out data analysis and interpretation. NHK, JHH, JYP, PGC, WBP, ESK, SWP, KUP, HBK, NJK, ECK and MDO carried out data interpretation. YHJ and KHS drafted the manuscript. All authors have read and approved the final manuscript.

## Pre-publication history

The pre-publication history for this paper can be accessed here:

http://www.biomedcentral.com/1471-2334/12/239/prepub
